# Long-Term Effects of Obstetric Fistula on the Overall Quality of Life among Survivors Who Had Undergone Obstetric Fistula Repair, Central Gondar Zone, Northwest Ethiopia, 2020: A Community-Based Study

**DOI:** 10.1155/2022/6703409

**Published:** 2022-02-08

**Authors:** Getie Lake Aynalem, Belayneh Ayanaw Kassie, Chernet Baye, Animut Tagele Tamiru, Kiber Temesgen Anteneh, Aster Berhe, Wagaye Fentahun, Tibeb Zena Debele, Birhanu Wubale Yirdaw, Bayew Kelkay Rade, Mihretu Molla Enyew

**Affiliations:** ^1^School of Midwifery, University of Gondar, Gondar City, Ethiopia; ^2^Department of Gynecology/Obstetrics, University of Gondar, Gondar City, Ethiopia; ^3^UNFPA, Addis Ababa City, Ethiopia; ^4^UNFPA-Supported Maternal Health Project, University of Gondar, Gondar City, Ethiopia

## Abstract

**Introduction:**

Childbirth is a special time in the lives of women and families at large. It can also be a time of great tragedy. International reports show that, annually, more than 500,000 women die from pregnancy and childbirth complications globally. For every woman who dies in childbirth, majorities remain alive, but scarred by permanent disabilities. Obstetric fistula is, without a doubt, the most severe of pregnancy-related disabilities.

**Objective:**

This research is aimed at assessing the long-term effects of obstetric fistula on the overall quality of life among fistula survivors in central Gondar zone.

**Methods:**

A community-based cross-sectional study was conducted among women who had undergone obstetric fistula repair, 1-4 years after the surgery, in the central Gondar zone. The participants were reached through appointments that were made by the researchers using census approach after having the participants' contact lists (specific residence and cell phone numbers), and research interviews have taken place at the respondents' home or residence using an adapted and validated tool. Data entry and analysis were done using Epi Info version 7 and SPSS version 20, respectively.

**Results:**

A total of 182 fistula survivors were interviewed giving a 94.8% response rate. This study indicated that 84.1% (95% CI: 78.8, 89.4) of respondents had a poor overall quality of life. Maternal age (>30 years) (AOR = 3.8, 95% CI: 2.6, 12.3), marital status (divorced survivors) (AOR = 2.7, 95% CI: 1.3, 8.5), and urinary incontinence (AOR = 1.9, 95% CI: 2.4, 11.2) were positive predictors for poor overall quality of life. The majority of fistula survivors, 82.4%, were stigmatized which could make reintegration into the community challenging for them. Healthcare providers have to implement counseling to women for social reintegration and the possibility of gainful societal activities after repairing.

## 1. Introduction

Pregnancy is a period which is unique for the lives of women and families. However, it can also be a period of developing huge complications including death. Globally, more than 500,000 women die from pregnancy complications and childbirth annually. These deaths affect families and leave children without their guardians (protectors). For every woman who dies in childbirth, many more remain alive, but scarred by permanent disabilities. Obstetric fistula, which is almost exclusively occurring in developing countries, is, without a doubt, the most severe of the pregnancy-related disabilities [1].

Obstetric fistula is an abnormal communication between the two epithelial tissues (genital and urinary tracts, vesicovaginal fistula; genital tract and rectum, rectovaginal fistula). The causes for obstetric fistula are different in high- and low-income countries. Even though the occurrence of obstetric fistula in high-income countries is very rare, it may be mainly due to radiation damage, surgical complications, congenital anomaly, and malignancy [2]. But it occurs mainly due to neglected prolonged obstructed labor (OL) in low- and middle-income countries and continues to be profoundly hampering women's lives physically, economically, and psychosocially even after the repair [2, 3].

Low-income countries are the top ones to be affected by this devastating disability, obstetric fistula. In Sub-Saharan African countries, it is estimated that obstetric fistulas occur in 1 to 3 of every 1000 deliveries [2]. The World Health Organization (WHO) estimates, globally, more than 300 million women remain alive from pregnancy but suffer from short- or long-term complications of it, in which the top is obstetric fistula. According to the global estimation of obstetric fistula, there are 30,000-130,000 new fistula cases in Africa annually, 60,000 to 90,000 in Sub-Saharan Africa alone [2, 4]. It is also estimated that for every maternal death, there will be 20 women suffering from long-term disabling complications; obstetric fistula takes the lion share of this [1, 5].

There is a 5% worldwide estimation of OL which accounts for 8% of maternal deaths and almost all obstetric fistula cases. In developing countries, obstetric fistula is estimated to occur in 2% of OL. This OL can cause obstetric fistula if the mother encounters delay (delay for decision to seek care, delay for transportation after deciding for getting care, and delay for getting the obstetric emergency care after reaching the health institutions) for the emergency obstetric care like caesarean section. Youth and adolescent girls are particularly susceptible to this major cause of obstetric fistula, OL, because of pelvic immaturity [5].

There are also life-long disabilities caused by obstetric fistula. Women's productivity, community participation, socialization, household relationship, and quality of life can all be affected holistically. Guidelines and literatures show that obstetric fistula can be simply and successfully repaired, even though, it is a physical intervention [6]. The psychosocial, long-term quality of life in individual and interpersonal aspects, reintegration of the study participants into the community, productivity, and community participation of such cases are not yet investigated in Ethiopia, as to the best of the authors' search.

Even though it is limited, there is previous knowledge which shows the overall quality of women's life after fistula repair as compromised. A research in Malawi found that women who lived with repaired obstetric fistula are affected in community reintegration, childbearing, marital relation, and community activity participation aspects [5]. Obstetric fistula has also health and psychotropic negative effects even after repair. Stigma adhered with the obstetric fistula can enormously affect the quality of life of women. Scientific reports show that the consequences of obstetric fistula are within limits more than the evident medical condition itself [7, 8]. Divorce is another common consequence chiefly when women are with remaining incontinence after repair. Due to this, women may experience mental hurt including annoyance, misery, sadness, harshness, and shamefulness. As a result of this, there are fistula survivor cases that attempted suicide [9].

Successful surgical therapy alone does not complete the totality of obstetric fistula consequences: even though the hole is physically repaired, women who have had fistula may face ongoing depression, isolation from their families or communities, reduced opportunities for income, and other negative long-term outcomes [5].

Individuals with disability have the identical rights and equity chances as all other members of the community. Action plans have been endorsed by WHO as a joint position paper to suggest community-based rehabilitation (CBR) as a master plan for equalization of opportunities, rehabilitation, poverty reduction, and communal incorporation of individuals with disabilities [10]. For obstetric fistula survivor women, reintegration into the people requires redefinition of oneself and adjustment from being recognized as unclean, inferior, and unworthy to being perceived as clean, feminine, and energetic in the social and community lives [2]. But since obstetric fistula is common among rural women, clinical follow-up may be complicated because obstetric fistula survivor women returned to their rural homes making it difficult to contact them to assess the long-term impacts of fistula. The finding of this study, therefore, might be important for surgical programs dedicated to fistula repair to implement deep counseling of women for social reintegration and the possibility of gainful societal activities after the repair. Community-based inclusive development, the other name of CBR, has its branch office in University of Gondar, central Gondar zone, Amhara regional state. As this office has been implementing its master plans, the data from this research may be important as a baseline data for further research, one of CBR's objectives to achieve its endorsed action plan, on fistula survivors.

To the best of the authors' knowledge, the present study is the first in its kind and is aimed at assessing the overall long-term quality of women after they had undergone obstetric fistula repair in the University of Gondar comprehensive specialized hospital, central Gondar zone, Northwest Ethiopia.

## 2. Methods and Materials

### 2.1. Study Design, Setting, and Population

A community-based cross-sectional study was conducted among women who had undergone obstetric fistula repair in the University of Gondar comprehensive specialized hospital from 2016 to 2019, central Gondar zone, Northwest Ethiopia. According to the central Gondar zone labor and social affairs department 2010 report, the central Gondar zone has 17 districts and 443 (391 rural and 52 urban) kebeles (the smallest administrative unit). Each kebele has at least one health extension worker. There were a total of 192 women who had been sent to their home after the surgical repair of their obstetric fistula. Each woman had her own and her attendant's contact lists (her telephone number, her attendant's telephone number, her district, and her kebele) available in the hospital. The authors collected these contact lists from the hospital after submitting the ethical clearance and used to reach the study participants in their residence. Women who had fulfilled the eligibility criteria mentioned below (in Sample Size Determination) were our study population.

### 2.2. Sample Size Determination

Census technique was used to reach the study participants since the case was rare in nature (the authors had counted down 8 fistula cases in a month and have been told by the staff working in the fistula center that the average number of fistula cases could be from 7 to 8 per month). So, the total cases that investigators would have were likely to be less than the total kebeles in the central Gondar zone: that is, the feasibility issue may not be a concern. There were eligibility criteria for the selection as a study participant: (1) history of obstetric fistula repair in the University of Gondar comprehensive specialized hospital, (2) residence in one of the central Gondar zonal kebeles, (3) time after the repair elapsed from 1 to 4 years, and (4) participants who could be reached via one of their addresses. The participants were reached through the appointments that were made by the research assistants using their contact lists that were provided by the hospital, and the research interviews have taken place at their home or district of residence.

### 2.3. Data Collection Tools and Procedures

A structured, pretested, and interviewer-administered tool adapted from literatures [10–12] was used to collect data on basic demographic information, the experience and feeling of the respondents, and the moral and social support from their family, friends, and community after their obstetric fistula repair. This tool was contextualized by the authors after the detailed reviewing of the previous similar studies. Fistula surgeons and obstetricians were consulted for the tool contextualization. The questionnaire for the data was prepared in English, and the interviews were conducted in Amharic (local language) after translation from English to Amharic. A one-day training was given for data collectors and supervisors about the aim of the study, contents of the tool, and ways of collecting. The data collectors were the trained health extension workers and BSc Midwifery professionals after receiving the training. Nine health extension workers and four BSC Midwifery professionals as data collectors and three supervisors were involved in the data collection process. Data collectors, receiving the study participants' contact lists from the authors, had made appointments with the study participants using the telephone number and went home to home according to the appointments and interviewed there.

### 2.4. Data Quality Assurance

The quality of data was assured by proper designing and pretesting of the questionnaire on 5% of similar study participants in north Gondar zone and by giving training for the data collectors before the actual data collection. All authors and the consultant surgeons and obstetricians were the trainers. Every day after data collection, data were reviewed and checked for completeness, accuracy, and clarity by the supervisors and principal investigator and the necessary feedback was offered to facilitators. Data cleanup and cross-checking were done before the analysis.

### 2.5. Data Analysis Procedures

Epidemiological information (Epi Info) version 7 and SPSS version 20 were used for data entry and analysis, respectively. Descriptive analysis results were presented in the form of tables, figures, and text using frequencies and summary statistics such as mean, standard deviation, and percentage. Bivariate logistic regression analysis was used to determine the association of each independent variable with the outcome variable, and multivariable logistic regression analysis was also employed to adjust the influence of various independent variables (confounding effects) on the outcome variable. Odds ratio with 95% confidence interval was used to see the association between independent variables and dependent variable.

### 2.6. Measurements and Operational Definitions

- Overall quality of life: reports from the participants on the greatest burdens after surgical repair such as financial challenges, additional surgery, reproductive health and pregnancy/fertility desires, and relationship challenges like marital discord (unsupportive relationship) [6, 11]
- Good overall quality of life: when participants report none on the greatest burdens after the surgical repair of obstetric fistula [6, 11]
- Poor overall quality of life: when participants report at least one of the greatest burdens after the surgical repair of obstetric fistula [6, 11]

## 3. Results

### 3.1. Sociodemographic Characteristics of the Study Participants

A total of 182 participants were interviewed with the response rate of 94.8%. The mean age of the participants was 31.9 years with the SD of 6.8 years and 111 (61%) of the respondents' age group was in 30 and above years. More than three-fourths, 146 (80.2%), and a third, 52 (28.8%), of the study participants were Orthodox followers and private workers as to their religion and current occupational status, respectively. Regarding their marital status, the majority, 121 (66.5%), were divorced. Sixty-seven (36.8%) of the respondents' educational status was in the category of unable to read and write; 32 (17.6%) and 27 (14.8%) participants were from Chilga and Wogera, respectively, as to their districts. Three-quarters (75.3%) of the respondents were rural dwellers (Table 1).

### 3.2. Participants' Obstetric History

Two-fifths (40.7%) and less than a third (27.5%) of the study participants reported their gravidity history as 2 or 3 and greater than 3, respectively. More than half (51.6%) of the respondents reported that they did not have current alive child. Two-fifths (40.7%) of the participants did not have history of antenatal care follow-up for their last pregnancy (the pregnancy caused the obstetric fistula). Majority of the study participants (86.3%) had given birth through their birth canal for the last pregnancy (fistula-causing pregnancy) and nearly two-thirds (61%) of the study participants reported that the outcome of their last delivery was stillbirth. The mean age when the obstetric fistula occurred was 29.3 years with the SD of 6.7 years (Table 2).

### 3.3. Participants' Overall and Interpersonal Quality of Life

**Figure 1:**
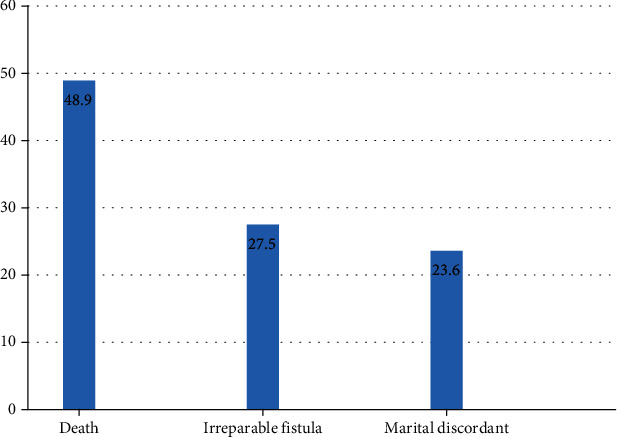
Percentage of fistula survivors' (*n* = 182) great concerns (fears) before the repair of their obstetric fistula, central Gondar districts, Northwest Ethiopia, 2020.

This study revealed that majority, 153 (84.1%) (95% CI: 78.8, 89.4), of fistula survivors were in the category of poor overall quality of life when measured by the indicators (financial challenges, additional surgery, and fear of reproductive health functionality including fertility and partner discordant (unsupportive relationship)). The greatest concern (fear) of the participants before the obstetric fistula repair was death (48.9%) followed by their fear of thinking that their fistula could be irreparable (27.5%) (Figure 1).

**Figure 2:**
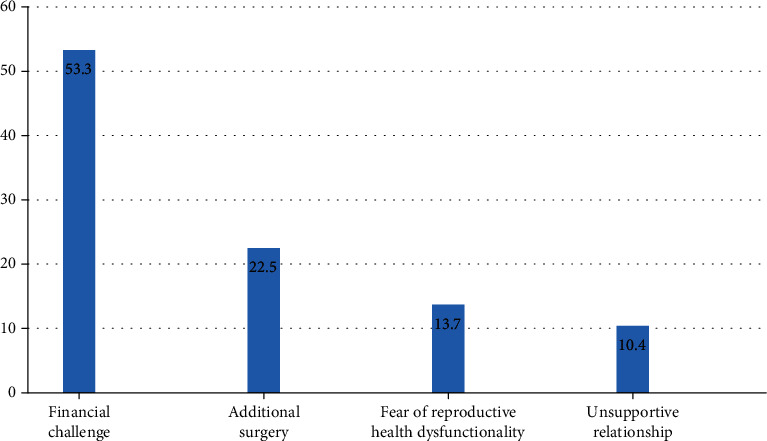
Percentage of fistula survivors' (*n* = 182) great burdens after the repair of their obstetric fistula, central Gondar districts, Northwest Ethiopia, 2020.

And the greatest concern after the repair of the obstetric fistula was financial challenge (53.3%) followed by fear of dysfunctional reproductive health including infertility (22.5%) (Figure 2).

Majority of the study participants, 151 (83%), had reported that their quality of life after the repair of the fistula was improved when compared with the quality of life before the repair.

Thirty-nine (21.4%) of the respondents reported that they had still urinary incontinence while walking, sitting, and lying. When stigmatization is concerned, majority of the participants, 150 (82.4%), had reported that they were stigmatized (dishonored). More than half (52.7%) of fistula survivors reported that they did not have desires to be pregnant. Half, 91 (50%), of the respondents had reported that they counseled women having similar cases to get treatment. One hundred twenty (96%) (*n* = 125 (divorced participants)) of our study participants reported out that the reason for their divorce was obstetric fistula (Table 3).

### 3.4. Factors Positively Associated with Overall Quality of Life of Fistula Survivors

- In the crude bivariable analysis: marital status of fistula survivor, urinary incontinence, residence, educational status, age of the woman, and stigmatization were variables significantly associated with the overall quality of fistula survivors
- In adjusted logistic regression: marital status, urinary incontinence, and age of the participants were variables positively associated with the overall quality of life

Those women who were divorced were 3 times more likely to have poor overall quality of life when compared with those who were married (AOR = 2.7, 95% CI: 1.3, 8.5).

Fistula survivors who were with urinary incontinence were 2 times more likely to have poor overall quality of life when compared with those who had no urinary incontinence (AOR = 1.9, 95% CI: 2.4, 11.2).

Study participants aged more than 30 were 4 times more likely to have poor overall quality of life when compared with those less than 20 years old (AOR = 3.8, 95% CI: 2.6, 12.3) (Table 4).

## 4. Discussion

This community-based study tried to describe the overall quality of life and its associated factors among women who had undergone fistula repair in the University of Gondar comprehensive specialized hospital, central Gondar zone districts, Northwest Ethiopia, 2020.

And the finding shows that majority of the study participants (84.1%) (95% CI: 78.8, 89.4) were categorized under poor overall quality of life even if they had their obstetric fistula repaired.

Nearly half, 48.4%, of fistula survivors reported that they had still urinary incontinence with different severities. In proportion, 19.2%, 7.7%, and 21.4% had it while coughing/straining, walking, or walking/sitting/lying, respectively. The finding is in line with the study done in Lilongwe, Malawi [6].

But this study has identified that fistula survivors had relatively lower proportions of incontinent women when compared with a study done in India that shows 100% of the study participants complained at least some urinary leakages [13]. The logic behind this difference could be the time duration after obstetric fistula repair. Waiting for at least one year after the repair was one of the criteria in this study but there was shorter time duration in India.

This study has revealed that majority of fistula survivors, 82.4%, were stigmatized (dishonored) by at least one of these: family, community, peer, or the whole three. This could make reintegration into the community challenging for fistula survivors. This finding is in line with the study done in Uganda [14]. However, the higher proportion of fistula survivors suffers from stigma in our study when compared with the study done in Lilongwe, Malawi (55%) [6]. The possible reason for the difference might be the sociodemographic differences between the two study populations. For example, in Lilongwe, Malawi, 55% were still married; but in this study, only 9.9% of the study participants were with their husbands. The authors have put this reason because marital status is a predictor variable for poor overall quality of life in our study.

The respondents in this study were asked, “If you get married (if unmarried), what is your desire for your future pregnancy?” More than half, 52.8%, of fistula survivors reported that they did not have any desire for future pregnancy. It shares similar findings with the study done in Lilongwe, Malawi [6]. However, according to the study which was done in southeastern Nigeria, relatively higher proportion of fistula survivors had reported their positive outlooks for their future pregnancy and became pregnant [12]. The difference might be due to the higher proportion of the study participants that was married in southeastern Nigeria, but the higher proportion of our study participants was divorced. Two-thirds, 66.5%, of fistula survivors in our study mentioned that the reason for the divorce was their obstetric fistula, be it before or after the repair.

Fistula survivors of our study shared their experiences with other women who sought fistula repair and the community at large. Half, 50%, of the participants reported that they had assisted women living in their surroundings and sought fistula repair, and 45.6% of the rest of the participants reported that they had been advocating for fistula prevention in the community at large. This may be a very important experience sharing because international and national reports showed that the burdens of obstetric fistula could be significantly reduced in the avoidance of treatment delay [11, 13]. The authors of this research recommend that healthcare providers in fistula centers should give health education for fistula cases so that they can continue advocating the prevention aspects in the community.

Maternal age group, >30 years, was one of the predictor variables for poor overall quality of life among the study participants. Fistula survivor cases whose ages were greater than 30 years were more likely to have poor overall quality of life when compared with those aged less than 20 years (AOR = 3.8, 95% CI: 2.6, 12.3). This finding is supported by the study done in Bangladesh in 2015 [15]. The reason might be that adolescents (young group) might get supports from their family. In addition, since they are young, their outlooks for their futurity might be better when compared with relatively older ones.

Marital status was also found to be one of the variables positively associated with the overall quality of life among fistula survivors. Those fistula survivor cases who were divorced were more likely to have poor overall quality of life when compared with the married ones (AOR = 2.7, 95% CI: 1.3, 8.5). This could be due to husbands' effort. Husbands might support their wives to reintegrate into the community and strengthen their interpersonal and social relationships so that might make the overall quality better.

Another variable that positively predicted poor overall quality of life among the study participants was urinary incontinence. Fistula survivors who were incontinent were more likely to have poor overall quality of life when compared with those who had no urinary incontinence (AOR = 1.9, 95% CI: 2.4, 11.2). It might be due to the fact that women who had urinary incontinence might have bad smell of the urine leakage that could make them isolated from family, peers, and community at large. This could possibly contribute for the poor overall quality of life.

### 4.1. Limitation of This Research

The finding of this research shares the drawback of a cross-sectional study design. Study participants might have been encountered with recall biases because the study's questionnaire contains points which could address the respondents' concerns before their obstetric fistula repair.

## 5. Conclusion

There was high proportion of fistula survivors with poor overall quality of life in the study setting. Women's age (above third decades), urinary incontinence, and divorce were variables positively affecting respondents' long-term quality of life. The authors recommend the local community health workers including health extension professionals to give a due attention for obstetric fistula survivors, counseling the survivors to check up their urinary incontinence and seek a care which could be one of the solutions to reduce their overall poor quality of life. The study also indicated that there was high stigmatization from family, peer, and community at large among the fistula survivors. Women who experienced stigma after obstetric fistula repair could be more likely to have problems in coping with reintegration into the community. Surgical programs should be dedicated to fistula repair, and healthcare providers should implement counseling to women for social reintegration and the possibility of gainful societal activities after repairing.

## Figures and Tables

**Table 1:** Sociodemographic characteristics of the study participants (*n* = 182), central Gondar districts, Northwest Ethiopia, June 2020.

Variable	Number (*n* = 182)	Percentage
Age		
<20	12	6.6
21-30	59	32.4
>30	111	61
Ethnicity		
Amhara	146	80.2
Kimant	36	19.8
District (woreda)		
Gondar Zuriya	20	11
Chilga	32	17.6
Alefa	17	9.3
Tegedie	18	9.9
Arimachiho	23	12.6
Wogera	27	14.8
Dembiya	18	9.9
Takussa	14	7.7
Belesa	13	7.1
Residence		
Rural	137	75.3
Urban	45	24.7
Current marital status		
Single	43	23.6
Married	18	9.9
Divorced	121	66.5
Religion		
Orthodox	146	80.2
Muslim	25	13.7
Protestant	11	6
Educational status		
Unable to read/write	67	36.8
Able to read/write	28	15.4
Primary education (1-8)	22	12.1
Secondary education (9-12) and above	65	35.7

**Table 2:** Obstetric history among fistula survivors (*n* = 182), central Gondar districts, Northwest Ethiopia, June 2020.

Variable	Number (*n* = 182)	Percentage
Ever had gravidity		
One	58	31.8
Two-three	74	40.7
>Three	50	27.5
Current alive children		
No child	94	51.7
1-3	57	31.3
>3	31	17
ANC for last pregnancy		
Yes	108	59.3
No	74	40.7
Mode of last delivery		
Vaginally	157	86.3
Caesarean section	25	13.7
Outcome of last delivery		
Alive	71	39
Dead	111	61
Age of fistula occurrence		
Less than 20	18	9.9
21-30	93	51.1
>30	71	39

**Table 3:** Overall and interpersonal quality of life among fistula survivors (*n* = 182), central Gondar districts, Northwest Ethiopia, June 2020.

Variable	Number (*n* = 182)	Percentage
Overall quality of life		
Poor	153	84.1
Good	29	15.9
Still urinary incontinence		
No	94	51.6
Yes (with cough/strain)	35	19.2
Yes (while walking)	14	7.7
Yes (while walking, sitting, and lying)	39	21.4
Stigmatization		
Yes	150	82.4
No	32	17.6
Stigmatization (*n* = 150)		
From family	3	2
From community	8	5.3
From peers	23	15.4
From all above	116	77.3
Future pregnancy desire		
No desire	96	52.8
In confusion	25	13.7
Had desire	61	33.5
Advocacy to others		
Advised to get treatment	91	50
Advised to prevent fistula	83	45.6
No advocacy	8	4.4
Divorce reasons (*n* = 125)		
Fistula itself	120	96
Out of the fistula	5	4

**Table 4:** Bivariable and multivariable analysis of factors associated with overall quality of life among fistula survivors (*n* = 182), central Gondar districts, Northwest Ethiopia, June 2020.

Variable	Poor overall quality of life		COR (95% CI)	AOR (95% CI)	*P* value
	No	Yes			
Age					
<20	3	9	1^∗^	1^∗^	
21-30	20	39	1.54 (1.2, 5.4)^∗∗^	1.2 (0.2, 6.3)	0.60
>30	80	31	7.74 (1.6, 18.3)^∗∗^	3.8 (2.6, 12.3)^∗∗∗^	<0.001
Marital status					
Married	12	8	1^∗^	1^∗^	
Single	28	15	1.2 (0.1, 4.1)	0.13 (0.1, 2.1)	
Divorced	89	31	1.9 (1.4, 11.3)^∗∗^	2.7 (1.3, 8.5)^∗∗∗^	<0.001
Urinary incontinence					
No	34	60	1^∗^	1^∗^	
With cough/strain	14	21	0.23 (0.2, 8.2)		
While walking	6	8	0.26 (0.1, 10.1)		
While walking, sitting, and lying	20	19	1.9 (2.5, 9.2)^∗∗^	1.9 (2.4, 11.2)^∗∗∗^	<0.05

Key: 1∗ = reference category, ∗∗ = significant in crude analysis, ∗∗∗ = significant in adjusted analysis.

## Data Availability

The dataset used is available from the corresponding author upon request.
